# Establishment of a Nipah Virus Disease Model in Hamsters, including a Comparison of Intranasal and Intraperitoneal Routes of Challenge

**DOI:** 10.3390/pathogens12080976

**Published:** 2023-07-26

**Authors:** Stephen Findlay-Wilson, Lucy Flett, Francisco J. Salguero, Ines Ruedas-Torres, Susan Fotheringham, Linda Easterbrook, Victoria Graham, Stuart Dowall

**Affiliations:** United Kingdom Health Security Agency (UKHSA), Porton Down, Salisbury SP4 0JG, UK; stephen.findlay-wilson@ukhsa.gov.uk (S.F.-W.); lucy.flett@ukhsa.gov.uk (L.F.); javier.salguero@ukhsa.gov.uk (F.J.S.); ines.ruedastorres@ukhsa.gov.uk (I.R.-T.); susan.fotheringham@ukhsa.gov.uk (S.F.); linda.easterbrook@ukhsa.gov.uk (L.E.); victoria.graham@ukhsa.gov.uk (V.G.)

**Keywords:** Nipah, model, intranasal, interperitoneal, challenge, efficacy

## Abstract

Nipah virus (NiV) is an emerging pathogen that can cause severe respiratory illness and encephalitis in humans. The main reservoir is fruit bats, distributed across a large geographical area that includes Australia, Southeast Asia, and Africa. Incursion into humans is widely reported through exposure of infected pigs, ingestion of contaminated food, or through contact with an infected person. With no approved treatments or vaccines, NiV poses a threat to human public health and has epidemic potential. To aid with the assessment of emerging interventions being developed, an expansion of preclinical testing capability is required. Given variations in the model parameters observed in different sites during establishment, optimisation of challenge routes and doses is required. Upon evaluating the hamster model, an intranasal route of challenge was compared with intraperitoneal delivery, demonstrating a more rapid dissemination to wider tissues in the latter. A dose effect was observed between those causing respiratory illness and those resulting in neurological disease. The data demonstrate the successful establishment of the hamster model of NiV disease for subsequent use in the evaluation of vaccines and antivirals.

## 1. Introduction

Nipah virus (NiV), from the genus Henipavirus in the family *Paramyxoviridae*, first emerged in Malaysia in 1998 [1]. Subsequent outbreaks since occurred in Bangladesh or India, beginning in 2001, almost on an annual basis [2]. Several species of fruit bat (*Pteropodidae* family) are thought to be reservoirs for Henipavirus, including those widely distributed across Australia, Southeast Asia, and Africa [3,4,5], thus maintaining a permanent risk of new outbreaks [6].

There are two main strains of NiV, designated NiV-Malaysia (NiV-M) and NiV-Bangladesh (NiV-B). Due to the different characteristics of human epidemiology, with the outbreak in Malaysia and Singapore having a case fatality rate (CFR) of approximately 40% [1], whereas in Bangladesh this is up to 92% in individual outbreaks [7], it is hypothesised that there may be differences in the pathogenicity of these strains. These strains were therefore compared in hamsters, including via the oronasal route [8], and show similar pathogenic traits, and even some delay of disease kinetic with the Bangladesh strain [9]. Therefore, it is likely that the different CFRs are due to other extrinsic factors. In Malaysia, the main exposure was through close contact with pigs [10], whereas in Bangladesh, exposure was reported through consumption of NiV-contaminated date palm sap [7,11] or direct contact from infected patients [12].

With a lack of established antivirals and vaccines available for use in humans, alongside a high CFR and human-to-human transmission, NiV is recognised as having the hallmarks of a pathogen with pandemic potential [13]. The availability of well-characterised animal models is essential for fulfilling the critical needs of the pre-clinical in vivo evaluation of potential interventions for human use, especially under the auspices of the Animal Efficacy Rule implemented by the U.S. Food and Drug Administration in 2002, which applied to the development of medical countermeasures where human efficacy studies are not possible or ethical, such as those caused by highly emerging pathogens such as NiV [14]. Therefore, animal studies are critical for the development of NiV vaccines and other medical interventions [15].

Syrian hamsters were well characterised as a susceptible model for NiV infection and disease progression [9,16,17,18,19,20,21], reproducing the hallmarks of respiratory and neurological clinical disease observed in humans [22,23]. Two main routes of challenge are used in NiV: intranasal (i.n.) and intraperitoneal (i.p.). Arguments for i.n. include a closer resemblance to a potential natural route of exposure to NiV in humans [20]. The i.p. challenge route, however, demonstrates a more uniform disease progression and readily allows for subtle difference to be elucidated, which may not be detectable in a less uniform infection route such as i.n. [9]. In addition, the disease progression after NiV challenge was shown to be affected by challenge dose, with high doses resulting in respiratory disease, whereas neurological signs and a more systemic spread of virus is observed with low doses [16].

In the hamster model, a variation in the lethal dose of NiV that causes 50% mortality (LD_50_) is observed. After the i.n. challenge, it was reported as being <1 TCID_50_ [16] to 47,000 pfu [21]. This may be due to challenge volumes delivered (100 μL vs. 30 μL) affecting the initial dispersion of the virus to the respiratory tract. In addition, there is a discrepancy between the main clinical disease manifestations, with one showing doses of 10^3^–10^6^ pfu being dominated by neurological signs [21] and another using similar doses of 10^5^ TCID_50_ being more dominated by respiratory signs [16].

With NiV being listed as a priority pathogen by the Coalition for Epidemic Preparedness Innovations (CEPI), the WHO Blueprint, and the UK Vaccine Network [24], it is widely recognised as being liable to cause future outbreaks. Therefore, the expansion of facilities able to assess interventions and study pathogenesis is required. Due to variations in model parameters in published reports, when establishing the hamster NiV model at the UK Health Security Agency (UKHSA) we compared different parameters, such as the route of infection and viral dose.

## 2. Materials and Methods

### 2.1. Ethical Statement

All experimental protocols with animals were undertaken according to the United Kingdom Animals (Scientific Procedures) Act 1986, with studies conducted under the authority of a UK Home Office-approved license. The experimental protocols were approved by ethical review at the UK Health Security Agency by the Animal Welfare and Ethical Review Body (AWERB) (Approval Code: PPL P82D9CB4B). This research is reported in accordance with the ARRIVE guidelines (https://arriveguidelines.org, accessed on 1 June 2023). Prior to the start of the study, humane clinical endpoints were set that consisted of 20% weight loss compared to baseline; inactivity/immobility; neurological signs; or on the advice of severe disease from the Named Animal Care and Welfare Officer (NACWO).

### 2.2. Animals

Golden Syrian hamsters (*Mesocricetus auratus*) were obtained from a UK home office accredited supplier (Envigo, Bicester, UK). For the first dose-ranging study (Study A), 24 female hamsters were used within two age ranges: 5–10 weeks and 18–19 weeks. Groups of *n* = 6 were challenged i.n. with 10^6^ and 10^5^ TCID_50_ and *n* = 4 challenged i.p. with 10^5^, 10^4^, or 10^3^ TCID_50_ NiV; with the different age groups split across the studies. For the second study (Study B) on dose confirmation, further dose reduction and pathogenesis, 16 male and 16 female hamsters were used, aged 6–11 weeks. Groups of *n* = 12 were challenged with 10^5^ TCID_50_ and *n* = 4 with 10^4^ TCID_50_ via the i.n. route. Groups of *n* = 12 were challenged with 10^3^ TCID_50_ and *n* = 4 with 10^2^ TCID_50_ via the i.p. route. Food and water were available *ad libitum*, with environment enrichment included in cages.

### 2.3. Virus

NiV (Malaysian strain; GenBank no. AF212302) was kindly provided by the Special Pathogens Branch of the Centers for Disease Control and Prevention, Atlanta, USA. Virus was propagated and titrated on VeroE6 cells (European Collection of Cell Cultures, Salisbury, UK) grown using Dulbecco’s minimal essential medium (DMEM; Gibco, Paisley, UK) supplemented with 2% fetal bovine serum (Gibco, Paisley, UK) at 37 °C. All infectious work was performed in a class III biological safety cabinet line in the containment level (CL) 4 laboratory at UKHSA, Porton Down.

### 2.4. Virus Challenge

Virus was diluted in sterile phosphate buffered saline (PBS) (Gibco, Paisley, UK) to achieve the relevant concentration for the challenge dose.

For i.n. delivery, the virus was instilled in a volume of 100 μL per nostril. For i.p. delivery, the virus was injected in a volume of 200 μL. Challenge was given under isoflurane sedation and animals monitored until a full recovery from sedation was observed.

### 2.5. Clinical Observations

Throughout the study, clinical signs were recorded at least twice a day by experienced husbandry and animal welfare staff. At the same time each day (07:00–09:00) animals were also weighed and had temperatures recorded via an implantable ID/temperature chip (idENTICHIP with Bio-Thermal, MSD Animal Health, Milton Keynes, UK). Clinical signs of disease were assigned a score based on the following criteria: 0, healthy; 1, behavioural change, eyes shut; 2, ruffled fur; 3, wasp-waisted, arched back, dehydrated; 5, laboured breathing; 8, ataxia; and 10, immobility, neurological signs, and paralysis. A cumulative score combining all observed signs was then assigned for each animal at that time point.

### 2.6. Necropsy Procedures

Hamsters were anaesthetised with isoflurane and then given an overdose of sodium pentobarbital at the scheduled end of the study (21 days post-challenge) or upon meeting humane clinical endpoint criteria. A necropsy was performed immediately after confirmation of death. A sample of blood was collected into animal blood RNAprotect tubes (Qiagen, Manchester, UK), and a sample of brain, liver, lung, and spleen into a dry tube. These were stored at −80 °C for viral RNA analysis. The remainder of the brain, liver, lung, and spleen was collected into histology pots containing 10% neutral-buffered formalin for fixation by immersion and further histopathological analysis.

### 2.7. Quantification of Virual Loads by RT-qPCR Preparation

Tissue samples for viral RNA analysis were weighed, resuspended in 1.5 mL PBS, and homogenised through a 400 μm mesh in a Netwell plate (Corning/Fisher Scientific, Loughborough, UK). Then, 200 μL of tissue homogenate or blood was transferred to 600 μL RLT buffer (Qiagen, Manchester, UK), and after at least 10 min, 600 μL 70% isopropanol was added to each sample. Samples were then transferred from the CL-4 laboratory to a CL-3 laboratory where contents were transferred to new tubes for RNA extraction outside of containment. Tissues were further homogenised through a QIAshredder (Qiagen, UK) at 16,000× *g* for 2 min and RNA was extracted by KingFisher Flex automatic extraction using the BioSprint 96 one-for-all veterinary kit (Indical, Leipzig, Germany) as per the manufacturer’s instructions. RNA was eluted in 100 μL AVE buffer (Indical, Leipzig, Germany). Samples were analysed by RT-PCR using the TaqMan Fast Virus 1-Step Master Mix RT-PCR kit (ThermoFisher, Loughborough, UK) with the fast-cycling mode and primers/probes targeting the N gene of the Nipah virus Malaysian strain (NCBI Reference Sequence: NC_002728.1) (adapted from [25]). Quantification of viral load was determined using a 10-fold serial dilution of the NiV N gene in vitro transcript [2.0 × 10^6^ to 2.0 × 10^0^ copies µL^−1^] (Integrated DNA Technologies, Leuven, Belgium).

### 2.8. Histopathological Analysis

Tissue samples were fixed by immersion in 10% neutral-buffered formalin for at least 3 weeks before being processed routinely into paraffin wax, and 4 μm sections were cut and routinely stained with haematoxylin and eosin (H&E). All slides were digitally scanned using a Hamamatsu S360 digital slide scanner (Hamamatsu Photonics K.K., Shizuoka, Japan) and examined using ndp.view2 software (Hamamatsu Photonics K.K., v2.8.24).

In the lung, the severity of lesions (broncho-interstitial pneumonia) was recorded. The presence of inflammatory infiltrates was recorded in the liver and spleen, together with the presence of lymphoid depletion in the latter. In the brain, the presence of meningitis and perivascular cuffing was also evaluated and scored. For each parameter, a semiquantitative score was given as 0 = within normal limits; 1 = minimal; 2 = mild; 3 = moderate; and 4 = marked/severe. For the spleen, the sum of inflammatory infiltrates and the level of lymphoid depletion was considered the final score, with 8 being the maximum possible score.

In addition, samples were stained using the in situ ybridization RNAscope technique to identify NiV RNA. Briefly, slides were pre-treated with hydrogen peroxide for 10 min (room temperature), target retrieval for 15 min (98–101 °C), and protease plus for 30 min (40 °C) (Advanced Cell Diagnostics, Newark, USA). A NiV-specific probe (Cat No. 439258, Advanced Cell Diagnostics) was incubated with the tissues for 2 h at 40 °C. Amplification of the signal was carried out following the RNAscope protocol using the RNAscope 2.5 HD Detection Kit–Red (Advanced Cell Diagnostics). Likewise, slides were digitally scanned and evaluated with the Nikon NIS-Ar software (Nikon, Praha, Czech Republic) in order to quantify the presence of viral RNA (percentage area positively stained) by digital image analysis.

Histopathology and in situ hybridisation RNAscope technique were carried out in a ISO9001:2015 and GLP-compliant laboratory and evaluation was performed by qualified veterinary pathologists blinded to the study groups.

### 2.9. Statistical Analysis

Statistical analyses were performed using Minitab, version 16.2.2. (Minitab Inc., Chicago, USA). For comparison of survival, nonparametric distribution analysis (right censoring) was undertaken on Kaplan–Meier plots. A non-parametric Mann–Whitney U statistical test was applied to ascertain significance between groups. A significance level ≤ 0.05 was considered statistically significant.

## 3. Results

### 3.1. Dose Determination Study

#### 3.1.1. Clinical Parameters

**Figure 1:**
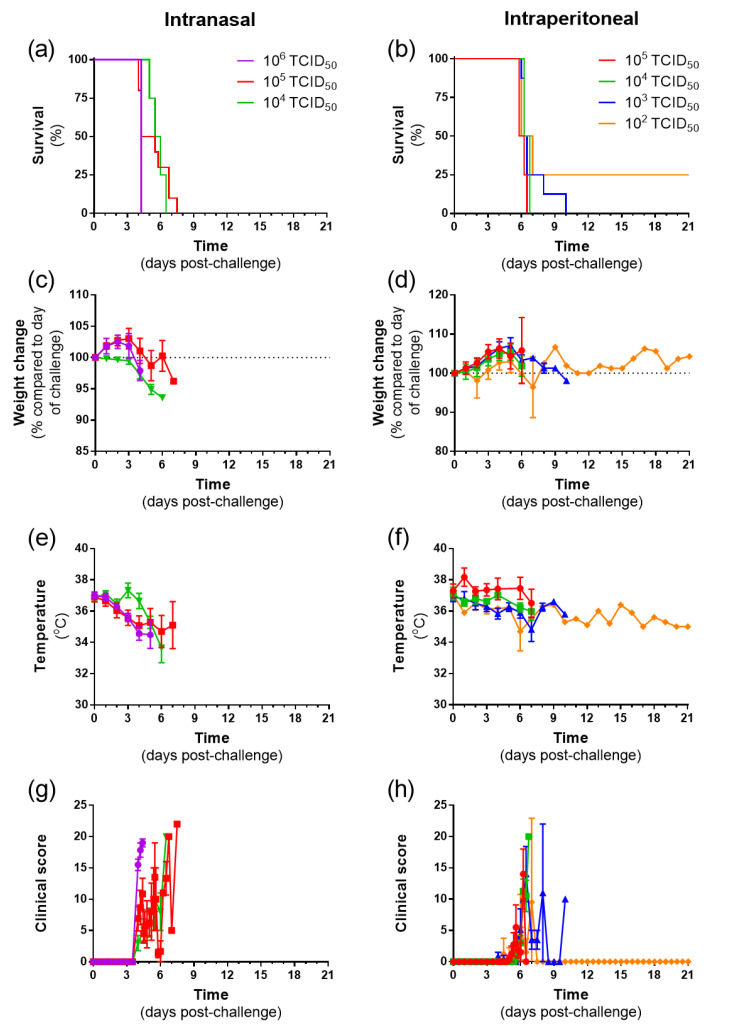
Clinical parameters from hamsters challenged intranasally and intraperitoneally with Nipah virus at different doses. (**a**,**b**) Kaplan–Meier survival plots. (**c**,**d**) Weight change compared to the day of challenge. (**e**,**f**) Temperature. (**g**,**h**) Clinical score. (**c**–**h**) Data show mean values with error bars denoting standard error from *n* = 6, 10, and 4 (10^6^, 10^5^, and 10^4^ doses, respectively; i.n. route) and *n* = 4, 4, 8, and 4 (10^5^, 10^4^, 10^3^, and 10^2^ doses, respectively; i.p. route).

To ascertain the susceptibility of golden Syrian hamsters to NiV infection, dose ranging studies were conducted. Study A used doses of 10^6^ and 10^5^ for i.n. and 10^5^–10^3^ for i.p. challenge. Study B then used the previously used lower dose and a one-log-lower dose. Results from the two studies were combined (Figure 1). All animals challenged via the i.n. route met humane endpoints, whereas one animal survived until the scheduled end of the study when i.p.-challenged with the lowest dose, 10^2^ TCID_50_ (Figure 1a,b). The clinical course of disease was accompanied by a loss in weight (Figure 1c,d). A drop in temperature was evident in animals i.n.-challenged, whereas temperatures were more stable in those challenged by the i.p. route (Figure 1e,f). Clinical signs were observed from day 3 post-challenge when delivered i.n., but were observed later in those that were i.p.-challenged (Figure 1g,h).

**Figure 2:**
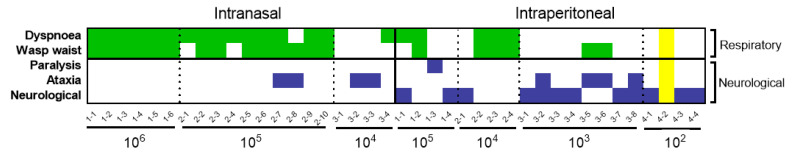
Heatmap representation of major clinical signs recorded at humane clinical endpoint. Green boxes, respiratory signs; blue boxes, neurological signs; and yellow boxes, animal did not meet humane clinical endpoint.

Animals met humane clinical endpoints based on the severity of the disease. These signs were divided into those associated with the respiratory system (dyspnoea and wasp waisted) and neurological signs (paralysis, ataxia, and neurological). Animals challenged via the i.n. route mainly exhibited respiratory signs, whereas those challenged via the i.p. route at high disease increasingly exhibited neurological signs (Figure 2).

#### 3.1.2. Direct Comparison of Intranasal and Intraperitoneal Inoculation Routes

**Figure 3:**
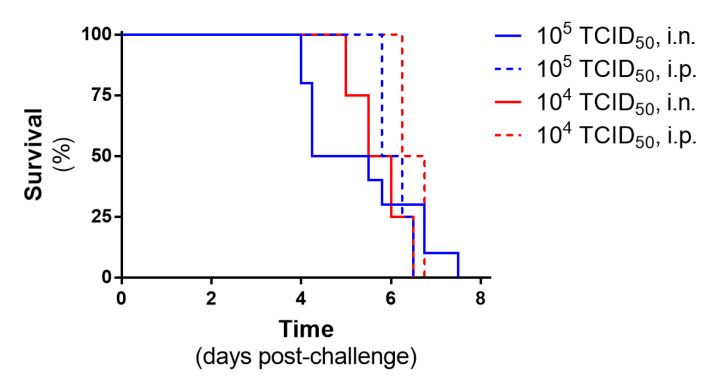
Comparison of i.n. and i.p. routes using the same challenge dose of Nipah virus. Survival plots show values from *n* = 10 and 4 (i.n. and i.p. routes, respectively; 10^5^ dose) to *n* = 4 and 4 (i.n. and i.p. routes, respectively; 10^4^ dose).

During the dose determination studies, challenge doses of 10^5^ and 10^4^ TCID_50_ were tested using both i.n. and i.p. routes. When these routes were compared, the survival graphs were similar, with all animals meeting humane clinical endpoints 4–8 days post-challenge (Figure 3). There were no discernible statistically significant differences in survival kinetics for either the 10^5^ or 10^4^ challenge doses (*p* = 0.910 and *p* = 0.065, respectively; log-rank survival analysis).

#### 3.1.3. Reproducibility of Challenge Dose in Two Independent Studies

**Figure 4:**
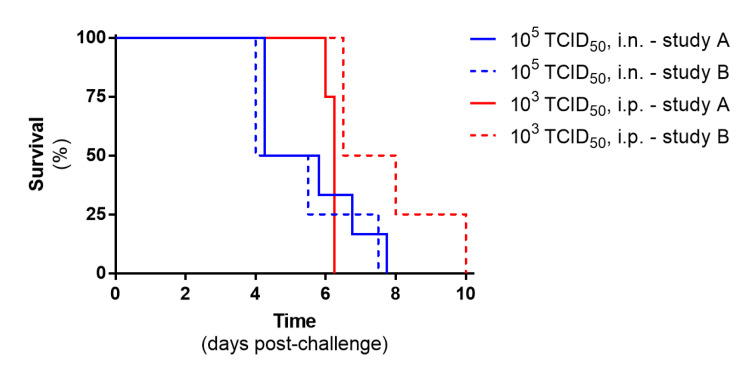
Reproducibility of survival plots across two independent studies using the same challenge doses and route of infection. Data show values from *n* = 6 and 4 (study A and B, respectively; i.n. route) to *n* = 4 and 4 (study A and B, respectively; i.p. route).

Challenge doses of 10^5^ and 10^3^ TCID_50_ were tested in Study A and Study B via the i.n. and i.p. routes, respectively. Survival plots show that these doses were consistent with all animals meeting humane clinical endpoints (Figure 4). The kinetics of the i.n. route were extremely similar, with no statistically significant effect seen (*p* = 0.462, Mann–Whitney test), whereas there was a delay in some animals meeting humane endpoints in the second study after i.p. challenge, which reached statistical significance (*p* = 0.010, Mann–Whitney test); however, the mean time to reaching humane endpoints only differed by 1.5 days (6.2 days for study A and 7.7 days for study B).

### 3.2. Pathogenesis Study

#### 3.2.1. Viral RNA Levels in Peripheral Blood and Tissues

**Figure 5:**
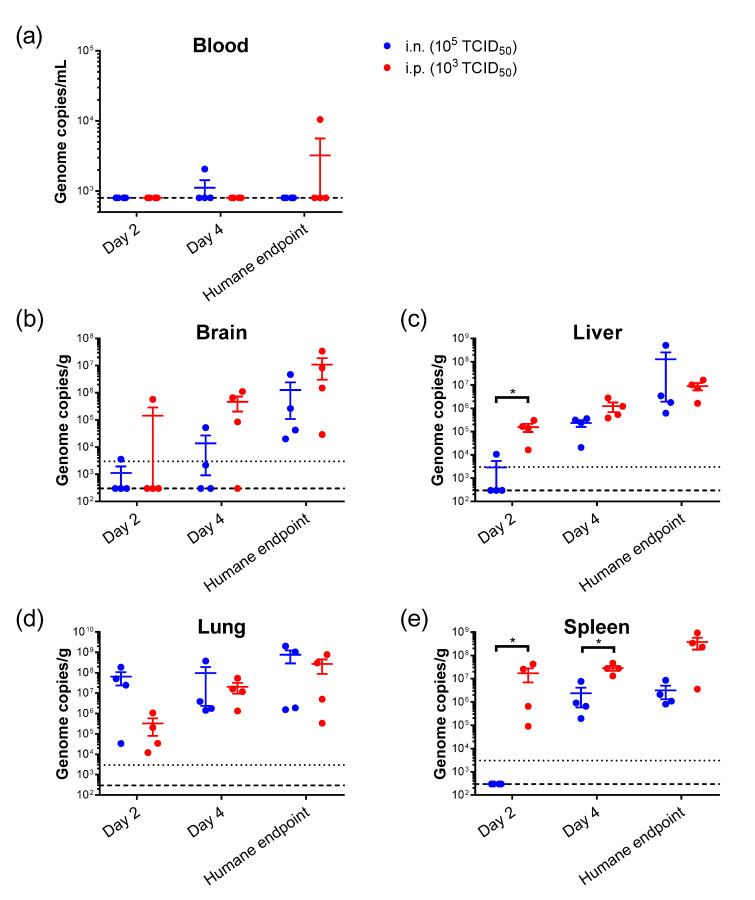
Viral RNA detection in hamsters challenged with NiV by either the i.n. or i.p. route and culled at scheduled timepoints. Viral RNA levels are shown from (**a**) blood, (**b**) brain, (**c**) liver, (**d**) lung and (**e**) spleen. Data points show values from individual animals with line and whisker plot denoting mean +/− standard error; *n* = 4 animals per timepoint. Dashed line, lower limit of detection; dotted line, lower limit of quantification. *, *p* < 0.05 (Mann–Whitney U test).

To ascertain key sites of viral infectivity, a pathogenesis study was conducted where hamsters challenged via the i.n. (10^5^ TCID_50_) or i.p. (10^3^ TCID_50_) routes were culled 2 and 4 days post-inoculation. Presence of viral RNA was assessed in the blood, brain, liver, lung, and spleen. Low levels of viral RNA were detected in the circulation in just 2 animals (Figure 5a). Within the brain, viral RNA was detectable in the majority of animals at day 4 and in all animals that met humane endpoints, irrespective of challenge route (Figure 5b). Viral RNA was observed in the liver at day 2 of all animals challenged via the i.p. route, which is statistically significant compared to the i.n. route (*p* = 0.0302, Mann–Whitney U test) (Figure 5c). In the lung, viral RNA was detected at all timepoints in all animals, with no statistical differences between challenge routes (Figure 5d). Results from the spleen show viral RNA in the i.p.-challenged animals at day 2, and by day 4—although detectable in the i.n. group—it remained significantly increased in the i.p. cohort (*p* = 0.0302, Mann–Whitney U test).

#### 3.2.2. Histopathological Analysis

**Figure 6:**
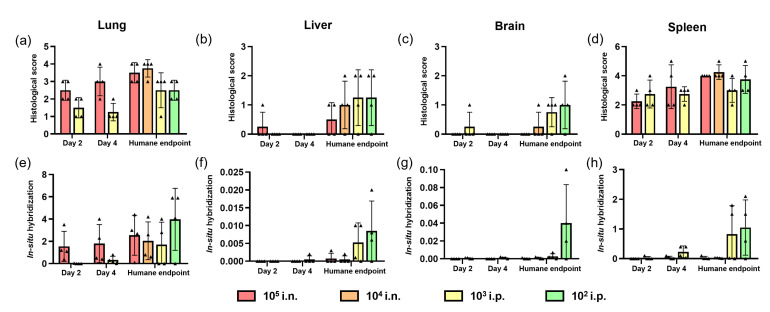
Quantitative readouts of histopathological score and in situ hybridisation results from samples collected at days 2 and 4 post-challenge and at humane endpoint. Histopathological score in (**a**) lung, (**b**) liver, (**c**) brain, and (**d**) spleen. Digital image analysis (percentage area positively stained) of in situ hybridisation in (**e**) lung, (**f**) liver, (**g**) brain, and (**h**) spleen. Data points show values from individual animals (black triangles) with columns and whisker plots denoting mean +/− standard error; i.n., intranasal route; i.p., intraperitoneal route; and *n* = 4 animals per timepoint.

**Figure 7:**
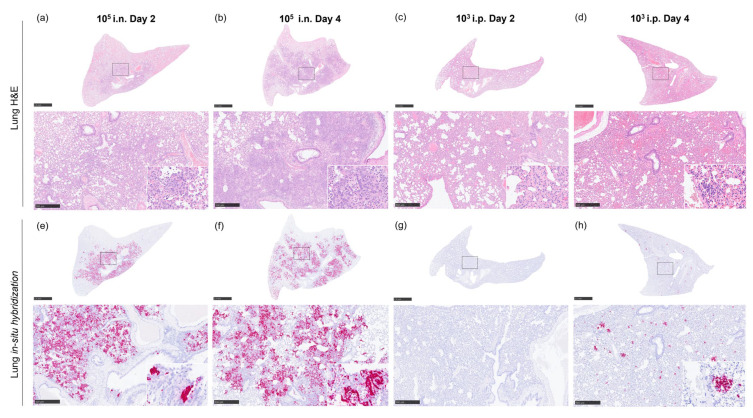
Representative histopathological images from lung NiV pathology (H&E) and in situ hybridisation from samples collected at days 2 and 4 post-challenge. (**a**) Lung section (H&E) from an i.n.-challenged animal with a 10^5^ TCID_50_ dose at day 2 post-challenge showing severe coalescing broncho-interstitial pneumonia. Inset shows thickening of alveolar walls, and infiltration of macrophages, neutrophils, and lymphocytes in septae and luminae. (**b**) Lung section (H&E) from an i.n.-challenged animal with a 10^5^ TCID_50_ dose at day 4 post-challenge showing severe coalescing broncho-interstitial pneumonia. Inset shows type II pneumocyte hyperplasia and mononuclear cell infiltrates. (**c**) Lung section (H&E) from an i.p.-challenged animal with a 10^3^ TCID_50_ dose at day 2 post-challenge showing mild interstitial pneumonia. Inset shows thickening of alveolar walls. (**d**) Lung section (H&E) from an i.p.-challenged animal with a 10^3^ TCID_50_ dose at day 4 post-challenge showing mild interstitial pneumonia. Inset shows a perivascular lesion within the parenchyma mainly composed of macrophages, with occasional lymphocytes and neutrophils. (**e**) In situ hybridisation from an i.n.-challenged animal with a 10^5^ TCID_50_ dose at day 2 post-challenge showing viral RNA in areas of severe broncho-interstitial pneumonia. Inset shows viral RNA in bronchiolar epithelial cells. (**f**) In situ hybridisation from an i.n.-challenged animal with a 10^5^ TCID_50_ dose at day 4 post-challenge showing viral RNA in areas of severe broncho-interstitial pneumonia. Inset shows higher magnification with abundant virus RNA within the parenchyma and airway epithelium. (**g**) In situ hybridisation from an i.p.-challenged animal with a 10^3^ TCID_50_ dose at day 2 post-challenge with no presence of viral RNA. (**h**) In situ hybridisation from an i.p.-challenged animal with a 10^3^ TCID_50_ dose at day 4 post-challenge showing viral RNA in multiple lesions. Inset shows viral RNA in a perivascular lesion; i.n., intranasal route; i.p., intraperitoneal route. Scale bars = 2.5 mm and 500 µm; insets = 100 µm.

**Figure 8:**
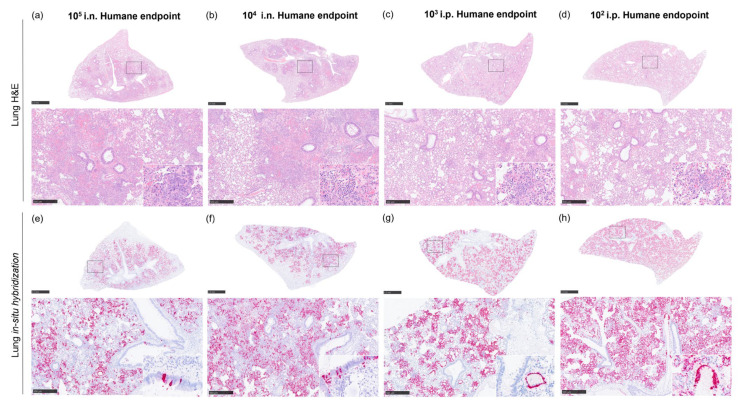
Representative histopathological lesions from lung NiV pathology (H&E) and in situ hybridisation from samples collected at humane endpoint. (**a**) Lung section (H&E) from an i.n.-challenged animal with a 10^5^ TCID_50_ dose showing severe coalescing interstitial broncho-pneumonia. Inset shows infiltrating macrophages infiltrate and type II pneumocyte hyperplasia. Alveolar macrophages, cell debris, and neutrophils are also present in alveolar spaces. (**b**) Lung section (H&E) from an i.n.-challenged animal with a 10^4^ TCID_50_ dose showing severe coalescing interstitial broncho-pneumonia. Inset shows lymphocytes, neutrophils and oedema in an alveolar space. (**c**) Lung section (H&E) from an i.p.-challenged animal with a 10^3^ TCID_50_ dose showing multifocal lesions. Inset shows macrophages and fewer lymphocytes and neutrophils within the lesion. (**d**) Lung section (H&E) from an i.p.-challenged animal with a 10^2^ TCID_50_ dose showing multifocal lesions. Inset shows macrophages and fewer lymphocytes and neutrophils within the lesion. (**e**) In situ hybridisation from an i.n.-challenged animal with a 10^5^ TCID_50_ dose showing viral RNA in areas of severe interstitial broncho-pneumonia. Inset shows viral RNA in bronchiolar epithelial cells. (**f**) In situ hybridisation from an i.n.-challenged animal with a 10^4^ TCID_50_ dose showing viral RNA in areas of severe brocho-interstitial pneumonia. Inset shows viral RNA in bronchiolar epithelial cells and parenchyma. (**g**) In situ hybridisation from an i.p.-challenged animal with a 10^3^ TCID_50_ dose showing viral RNA in multifocal lesions. Inset shows viral RNA in endothelial cells from blood vessels. (**h**) In situ hybridisation from an i.p.-challenged animal with a 10^2^ TCID_50_ dose showing diffuse expression of viral RNA within the lung. Inset shows viral RNA in endothelial cells from blood vessels and the parenchyma; i.n., intranasal route; i.p., intraperitoneal route. Scale bars = 2.5 mm and 500 µm; insets = 100 µm.

Histopathological changes were mainly observed in the lung from animals inoculated with i.n. route at day 2 and 4 post-challenge until reaching a humane endpoint (Figure 6a). In these animals, pulmonary lesions consisted of severe multifocal and coalescing areas of broncho-interstitial pneumonia characterised by necrosis of alveolar and bronchiolar epithelium, thickening of the alveolar walls by infiltrating macrophages, and to a lesser extent, lymphocytes and plasma cells (Figure 7a,b and Figure 8a). In most severe cases, neutrophils, cell debris, alveolar macrophages, and mucus plugs were observed within the alveolar, bronchiolar, and bronchial luminae (Figure 8b). In animals inoculated through i.p. route, moderate lesions were observed (Figure 6a), characterised by the presence of multifocal areas of pneumonia, mainly located around blood vessels or airways, infiltrating into the lung parenchyma (Figure 7d and Figure 8c,d). These lesions were mainly composed of macrophages and fewer lymphocytes, plasma cells, and neutrophils (Figure 7d and Figure 8c,d, insets).

Higher presence of viral RNA (RNAScope ISH) was observed in the lung of i.n.-challenged animals compared the i.p. route at day 2 and 4 post-challenge (Figure 6e). In i.n.-challenged animals, the viral RNA was located in areas of severe broncho-interstitial pneumonia (Figure 7e,f), whereas in those i.p.-inoculated, the virus was located within the multifocal lesions (Figure 7h). At humane endpoint, a similar viral RNA presence was observed in the lung from different challenged groups, with higher percentages of positively stained areas in those from animals inoculated with 10^2^ TCID_50_ via i.p (Figure 6e). Moreover, in i.n.-challenged animals, viral RNA was also present in bronchiolar epithelial cells (Figure 7e and Figure 8e,f, insets), while in i.p.-challenged animals, virus was also observed in endothelial cells from blood vessels (Figure 8g,h, insets).

In the liver, no significant lesions were observed at day 2 and 4 post-challenge (Figure 6b). However, a higher histopathological score was observed in tissues from animals euthanised at humane endpoint, mainly in those challenged with 10^3^ and 10^2^ TCID_50_ through i.p. route, which was correlated with the presence of viral RNA by in situ hybridisation (Figure 6b,f). Viral RNA was located within the liver sinusoids, Kupffer cells, and endothelial cells from hepatic blood vessels (data not shown).

**Figure 9:**
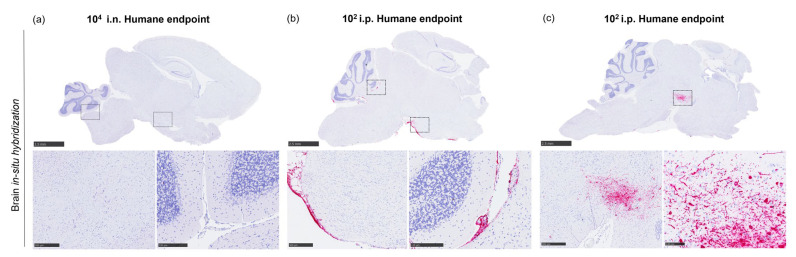
Representative RNAScope in situ hybridisation images from brain samples collected at humane endpoint. (**a**) In situ hybridisation from an i.n.-challenged animal with a 10^4^ TCID_50_ dose not showing viral RNA expression. (**b**) In situ hybridisation from an i.p.-challenged animal with a 10^2^ TCID_50_ dose showing viral RNA in meningeal inflammatory infiltrates and blood vessels. (**c**) In situ hybridisation from an i.p.-challenged animal with a 10^2^ TCID_50_ dose showing viral RNA in neurons and neuropil within the mid-brain; i.n., intranasal route; i.p., intraperitoneal route. Scale bars = 2.5; insets = (**a**,**b**), 500 µm and 250 µm; and (**c**), 500 µm and 100 µm.

In the brain, more striking lesions were found in i.p. challenge animals at the end of the study, mainly with a 10^2^ TCID_50_ dose (Figure 6c), the presence of meningitis (infiltration of mononuclear cells within the meninges) being the main observation found. In this group, high expression of viral RNA was observed in the areas of lesions in comparison with the rest of the inoculated groups (Figure 6g and Figure 9). Viral RNA was mainly detected in inflammatory cells within the thickened meninges and endothelial cells from blood vessels (Figure 9b). Additionally, neurons and neuropil within the mid-brain regions showed expression of viral RNA in this group (Figure 9c).

In the spleen, similar histopathological scores were observed at 2 and 4 days post-challenge and at humane endpoint, with values slightly higher in the animals euthanised at humane endpoint (Figure 6d). Diffuse expression of viral RNA was observed throughout the splenic parenchyma, mainly in the red pulp of these animals (data not shown), and mostly in tissues from i.p. route-inoculated animals at the end of the study (Figure 6h).

## 4. Discussion

In our study, doses resulting in all animals meeting humane clinical endpoints were observed. This conflicts with other reports, where either an LD_50_ only is reported so it is not possible to know if all animals succumb to severe disease [16], or where even at higher doses some survival is still observed. Using a dose of 10^5^ and 10^7^ TCID_50_ via the i.n. route, 62.5 and 33.3% of challenged animals survived, albeit using a recombinant NiV strain [26]. Models where diseases cause all untreated animals to meet humane endpoints are beneficial in the conduction of efficacy studies against severe disease in humans as they exhibit a clinically relevant endpoint of protection [27]. Whilst we ascertained the lowest lethal dose for i.p., we did not with the i.n. route (even whilst using the lowest dose tested of 10^4^ TCID_50_). Of note, where a 10-log dilution series was conducted with an i.p. challenge route, findings align with our results, where survival is seen with doses of 10^2^ TCID_50_ and lower; but at higher doses, all animals meet endpoint criteria [9]. Following i.p. challenge, the LD_50_ was reported as being 68 TCID_50_ [9].

We observed that with lower doses, animals were more likely to exhibit neurological signs in line with other reports [16]. However, other studies found no correlation between dose and subsequent respiratory or neurological disease progression [17] or the occurrence of both neurological signs as well as dyspnoea in the final stages of disease [21]. These results are similar to other viral pathogens, such as the influenza virus, where lower challenge doses resulted in differential disease outcomes [28].

We showed no difference in survival between the two different challenge routes, which is in line with other reports [16]. The i.p. route appears more uniform compared to the i.n. route [21], and is the main reported route of challenge for efficacy testing of NiV vaccine candidates [29,30,31,32,33,34,35,36,37] and antivirals [38]. Whilst we did not assess aerosol exposure, it was reported that this route does not change the course of disease compared to i.n. inoculation at similar doses [17]. With the aerosol route, only the highest dose (10^5^ pfu) resulted in all animals meeting endpoint criteria, with doses 2 × 10^4^ pfu and lower resulting in animals surviving even after 40 days post-challenge [17].

Viral RNA levels measured by RT-PCR showed that in animals challenged via the i.n. route, the virus primarily targeted the lung before then spreading to the spleen and liver, and finally the brain. Using the i.n. route, others also showed efficient virus replication in the respiratory tract [16]. Given the challenge routes used (i.n. and i.p.), the observation of respiratory virus involvement is not surprising. Given the route of exposure via consumption of date palm sap in the outbreak in Bangladesh [7,11], further work could look into virus infectivity and subsequent spread following ingestion. The finding of the virus and histopathological lesions in the spleen align with findings in fatal cases of NiV infection in humans [22]. Our results complement those of Munster et al., where hamsters were i.n.-challenged with the same dose, 10^5^ TCID_50_, and serially culled to assess progression from the nasal cavity to the central nervous system (CNS) [19]. Their results demonstrate entry into the CNS by day 4 post-challenge, in line with our observations of viral RNA presence in the brain at this timepoint. For animals meeting humane endpoints, RT-PCR data show little difference between the i.p. and i.n. route, showing that by this time, organ involvement is similar. However, at day 2 post-challenge, there was a clear distinction with viral RNA only being in the lung in i.n.-challenged animals, whereas dissemination to the liver, spleen, and brain was already evident in the i.p.-challenged group.

Interestingly, viral RNA in the blood was rarely detectable, which is a finding corroborated by others [16]. The dissemination of the virus throughout respiratory tissues was hypothesised to be due to physical spread through respiration or via the mucociliary apparatus [20]. Whilst RT-PCR provides evidence that the virus does not disseminate very efficiently via the hematogenous route, it may be that the level of detection was low and that some virus is present, which accumulates in the spleen due to one of its functions being to remove blood-borne pathogens via filtration [39,40]. Spread into the CNS was shown to be possible via olfactory nerves in both pig [41] and hamster [19] models. Importantly, we tested viral RNA from whole blood samples. This is due to its association with circulating cells, rather than being free in serum, due to binding onto leukocytes [42]. After the i.p. challenge of hamsters, NiV was shown to use this method of leukocyte capture and carry, without being productively infected, to transfer live viruses to other permissive cells [42]. Whilst the study was not designed to comprehensively assess the route of virus entry into the brain, the low levels of virus in the blood suggest it is unlikely to be via crossing the blood-brain barrier.

Histopathology results show lower levels of viral RNA detection compared to RT-PCR assay, especially in the liver. Similarly, despite the presence of viral RNA detected in the brain by RT-PCR at day 4 post-challenge in animals challenged via the i.p. route, changes were not observed with the histology score or RNAscope staining levels. This may be due to different limits of the detection of the assays, with histological and RNAscope assessment being limited to a small area of a tissue section, whereas RT-PCR was performed on a larger homogenised sample section. This observation was also highlighted in the analysis of SARS-CoV-2 viral RNA from challenged hamsters [43]. Another plausible explanation is that the RNAscope probe used here targets only the positive-sense RNA observed during virus replication [20].

Given the pandemic potential of NiV infections [44], particularly the well-documented human-to-human transmission events [12,45,46,47] and predisposition to cause nosocomial infection [48], the expansion of facilities able to model infection will assist in preparedness and strengthen the ability to respond to future outbreaks and assess the effectiveness of intervention strategies. The changing environment and resultant ecological pressures on the fruit bat reservoir altering foraging and behavioural patterns, owing to deforestation and urban development, may also enable NiV and similar Henipavirus infections to continue to emerge and re-emerge across a wide geographical area [49]. Whilst there are non-human primate models for NiV infection [50], the hamster model has advantages with the ease of procurement and reduced housing requirements, thus enabling studies to be fulfilled in numbers able to reach statistical power more efficiently [27].

## Data Availability

The data presented in this study are available on request from the corresponding author.
